# Identification and validation of a small molecule targeting ROR1 for the treatment of triple negative breast cancer

**DOI:** 10.3389/fcell.2023.1243763

**Published:** 2023-09-13

**Authors:** Shradheya R. R. Gupta, Tram M. Ta, Maryam Khan, Archana Singh, Indrakant K. Singh, Bela Peethambaran

**Affiliations:** ^1^ Molecular Biology Research Laboratory, Department of Zoology, Deshbandhu College, University of Delhi, New Delhi, India; ^2^ Department of Biology, Saint Joseph’s University, Philadelphia, PA, United States; ^3^ Department of Botany, Hans Raj College, University of Delhi, New Delhi, India; ^4^ Delhi School of Public Health, Institute of Eminence, University of Delhi, New Delhi, India; ^5^ Norris Comprehensive Cancer Center, Division of Medical Oncology, University of Southern California, Los Angeles, CA, United States

**Keywords:** breast cancer, TNBC, Ror1, kinase, MCF-10A, MDA-MB-231

## Abstract

**Introduction:** Breast cancer is the most common cancer in women, with roughly 10–15% of new cases classified as triple-negative breast cancer (TNBC). Traditional chemotherapies are often toxic to normal cells. Therefore, it is important to discover new anticancer compounds that target TNBC while causing minimal damage to normal cells. Receptor tyrosine kinase-like Orphan Receptor 1 (ROR1) is an oncofetal protein overexpressed in numerous human malignancies, including TNBC. This study investigated potential small molecules targeting ROR1.

**Methodology:** Using AutoDock Vina and Glide, we screened 70,000 chemicals for our investigation. We obtained 10 representative compounds via consensus voting, deleting structural alerts, and clustering. After manual assessment, compounds 2 and 4 were chosen for MD simulation and cell viability experiment. Compound 4 showed promising results in the viability assay, which led us to move further with the apoptosis assay and immunoblotting.

**Results:** Compound 4 (CID1261330) had docking scores of −6.635 and −10.8. It fits into the pocket and shows interactions with GLU64, ASP174, and PHE93. Its RMSD fluctuates around 0.20 nm and forms two stable H-bonds indicating compound 4 stability. It inhibits cell proliferation in MDA-MB-231, HCC1937, and HCC1395 cell lines, with IC_50_ values of approximately 2 μM to 10 μM, respectively. Compound 4 did not kill non-malignant epithelial breast cells MCF-10A (IC_50_ > 27 μM). These results were confirmed by the significant number of apoptotic cells in MDA-MB-231 cells (47.6%) but not in MCF-10A cells (7.3%). Immunoblot analysis provided additional support in the same direction.

**Discussion:** These findings collectively suggest that compound 4 has the potential to effectively eliminate TNBC cells while causing minimal harm to normal breast cells. The promising outcomes of this study lay the groundwork for further testing of compound 4 in other malignancies characterized by ROR1 upregulation, serving as a proof-of-concept for its broader applicability.

## Introduction

Breast cancer stands as a prevalent form of cancer commonly diagnosed among women, roughly 10%–15% of new cases as triple-negative breast cancer (TNBC) (Siegel et al., 2020; Almansour, 2022). In the United States alone, over 297,000 new cases of invasive breast cancer are expected to be reported in 2023, positioning it as the second leading cause of cancer-related fatalities among women^1^. Notably, this particular subtype of breast cancer is characterized by its aggressive nature and histological feature of lacking hormone receptors. Consequently, traditional hormone-based treatments such as Tamoxifen or Docetaxel have limited effectiveness in combating this form of breast cancer (Ring and Dowsett, 2004; Lorizio et al., 2012). The current standard of care for TNBC consists of traditional chemotherapy and radiation, which are often highly toxic to healthy cells. Consequently, the therapy failure rates for TNBC are far higher than those for other breast cancer subtypes (Foulkes et al., 2010; Boyle, 2012; Fallahpour et al., 2017), which is critical for identifying novel anticancer compounds targeting TNBC while maintaining the health of normal tissues. There is a need to identify candidate drugs that target specific proteins that cause and promote cancer.

Among various proteins, Tumor-Associated Antigens (TAAs) are expressed differently in cancerous and non-cancerous cells. These include Receptor Tyrosine Kinases (RTKs), which have been identified as critical regulators of various cellular activities, including cell-to-cell communication, proliferation, motility, differentiation, and metabolism (Du and Lovly, 2018; Zhao et al., 2021). They are also linked to the development and progression of various cancers, making them important targets for cancer treatment (Kamrani et al., 2019; Ghaderi et al., 2020). The RTKs superfamily is divided into 20 subfamilies, each comprising 58 members (Rozen and Shohet, 2022). One member is ROR1, which belongs to the receptor tyrosine kinase-like Orphan Receptor (ROR) subfamily (Zhao et al., 2021).

ROR1 is an oncofetal receptor discovered in a human neuroblastoma cell line in 1992 (Zhao et al., 2021). It is conserved across species and is primarily expressed in the plasma membrane as a transmembrane receptor. It has an extracellular domain (Ig-like domain, Frizzled/Cysteine-rich domain, and Kringle domain), transmembrane (TM) domain, and intracellular domain (Pseudokinase/Kinase domain, Proline-rich domain, and two Serine/Threonine-rich domains) (Shabani et al., 2015; Nath et al., 2017). Except for early B-cell precursors, its expression decreases during fetal maturation and becomes virtually non-existent in most *postpartum* tissues (Kipps, 2022). ROR1, a cell surface protein, is expressed in various malignancies, such as mantle cell lymphoma (MCL), acute lymphoblastic leukemia (ALL), and specific types of lung, ovarian, colon, breast, pancreatic, and renal cancers (Bemani et al., 2022). Cancer progression is influenced by the EGFR-mediated and Wnt signaling pathways, which are interconnected through the PI3K/AKT/mTOR pathway ROR1 is associated with advancing aggressive phenotypes in breast cancer, including triple-negative breast cancer (TNBC) (Bemani et al., 2022; Nadanaka et al., 2022). Research revealed that ROR1 is significantly upregulated at the transcriptional and translational levels, and this upregulation correlates with larger tumor size, lymphatic metastasis, and advanced TNM (Tumor (T), Nodes (N), and Metastases (M)) stages (II and IV) (Zhang et al., 2012; Balakrishnan et al., 2017). Additionally, experimental downregulation of ROR1 has been shown to inhibit cell proliferation and induce apoptosis in breast cancer cells (Chien et al., 2016). Consequently, targeting ROR1 has emerged as a promising strategy for developing anticancer drugs.

Monoclonal antibodies, drug-antibody conjugates, chimeric antigen receptor T-cells (CAR-T cells), and small chemical inhibitors have been investigated for targeting ROR1. However, only cirmtuzumab, a monoclonal antibody, has been clinically tested (Zhao et al., 2021). ARI-1 has been shown to attach to ROR1’s Frizzled/CRD domain, preventing Wnt5a from connecting to ROR1 (Zhao et al., 2021). KAN043983 is a small oral molecule that inhibits Wnt5a-induced ROR1 phosphorylation in CLL cells (Ghaderi et al., 2020; Sheetz et al., 2020; Kipps, 2022). KAN0441571C, a second-generation drug with improved efficacy, was developed by the same lab (Zhao et al., 2021). However, whether these small oral molecules target ROR1 directly is still unclear (Kipps, 2022). Previously, we found that the naturally occurring tannin Beta-1,2,3,4,6-Penta-O-Galloyl-D Glucopyranose was the highest-scoring ligand (Nath et al., 2017), and strictinin, a molecule similar to Beta-1,2,3,4,6-Penta-O-Galloyl-D Glucopyranose, was found to block ROR1 and its related downstream signaling (Fultang et al., 2019). Despite these promising findings, no small-molecule inhibitor of ROR1 has yet advanced to clinical trials, primarily due to concerns regarding their potential toxicity to normal cells. Therefore, this study aimed to address this critical gap by identifying and validating novel small molecules that selectively target ROR1 on TNBC cell lines. To achieve this, an integrated approach combining *in silico* and *in-vitro* techniques was employed.

## Materials and methods

### Preparation of the protein and ligand library

The pseudokinase/kinase domain of the ROR1 protein, ID 6TU9, was retrieved from the PDB database (Sheetz et al., 2020). The structure has four missing sections, generated using the MODELLER tool. Before docking, the structure was prepared using the AutoDock GUI tool and saved in PDBQT format. We selected and downloaded bioassay compounds used as kinase inhibitors, ROR family inhibitors, and ATP competitors from the PubChem database independent of their activity. Compounds with duplicate CIDs were removed using Microsoft Excel, and the list was uploaded to the PubChem database to download the compounds in SMILES format. For docking with AutoDock Vina, we converted the compounds into the PDBQT format with a protonation state of pH 7.4 using the OpenBabel tool.

### Docking and consensus voting

AutoDock Vina uses a Linux environment for docking. The program employed a gradient optimization approach for docking with random seeds. Random seed provides slightly different results each time the process is run on the same inputs, which aids in consensus voting (Plewczynski et al., 2011). Exhaustiveness was set to 8 for the initial docking and awarded a point to all compounds with a binding score of less than −10 kcal/mol. For the second run, we set exhaustiveness to 32 so that the tool could determine the best compound orientation with the lowest binding energy. All compounds with a binding score of less than −10 kcal/mol received a point. All compounds that obtained two points were docked again using Schrödinger’s Glide tool to increase the reproducibility of the results. Glide used a hierarchical clustering approach to determine the best hit (Friesner et al., 2004).

### Removing structural alerts and compound clustering

We used a Python script to eliminate compounds with high chemical reactivity, molecular fragments, and those activated with high chemical reactivity by human enzymes. With it, PAINS (Pan-Assay Interference Compounds) are also eliminated, which are physiologically active compounds that appear to be prospective therapeutic candidates during high-volume screening but are not (Baell and Nissink, 2018). The K-means clustering algorithm splits the filtered compounds based on the nearest structural mean value. The cluster size was set to 10, and representative compounds were chosen based on the lowest LogP value. Representative compounds were manually examined for ligand binding, pocket fitting, clashes, and noncovalent interactions with the protein.

### Molecular dynamic (MD) simulations

MD simulations can provide detailed information on protein-ligand interactions that cannot be obtained using methods such as docking (Hollingsworth and Dror, 2018). We performed MD simulations using GROMACS 2020.4 on the BRAF server of CDAC (Abraham et al., 2015). The force field used for protein preparation was CHARMM36-feb21. Bond order and residue name corrections were performed using the CGenFF web server for the ligand. The system solvation was carried out using the SPC water model in a cubic box. The steepest descent approach was used to process the solvated protein system for energy minimization for up to 25,000 steps or until the peak force (Fmax) did not exceed 1,000 kJ mol^−1^ nm^−1^. The NVT and NPT assemblies were assembled for 10 ns at 300 K and 1 atm pressure, respectively. The system temperature was first brought to equilibrium using a constant Number of Particles, Volume, and Temperature (NVT) ensemble. The pressure was then stabilized using the Number of Particles, Pressure, and Temperature (NPT) ensemble, which closely matched the experimental conditions. Finally, the production MD simulation was run for 200 ns to create trajectories. These trajectories determined the complex’s binding energies, fluctuations, and hydrogen bonds.

### Cell culture

The study used the TNBC cell line MDA-MB-231 (ATCC HTB 26) and the HCC1395 (ATCC CRL-2324TM. The MDA-MB-231 cells were grown according to ATCC recommendations in DMEM/F-12 supplemented with 10% FBS, insulin 10 μg/mL, non-essential amino acids, sodium pyruvate, penicillin 5,000 I.U/mL, and streptomycin 5,000 μg/mL. The non-malignant cell line used in this study was MCF-10A (ATCC CRL-10317TM) which was cultured in DMEM/F12K containing 5% horse serum, insulin (10 μg/mL), hydrocortisone (0.5 mg/mL), EGF (20 ng/mL), penicillin (100 U/mL), and streptomycin (0.1 mg/mL). HCC1395 (ATCC CRL-2324TM) and HCC 1937 (ATCC CRL-2336™), two other TNBC cell lines, were grown in RPMI with 10% FBS, penicillin (100 U/mL), and streptomycin (0.1 mg/mL). The cell cultures were maintained at 37°C and 5% CO_2_.

### Cell viability assays

An MTT assay (Thermo Fisher, Waltham, MA, United States) assessed breast cell proliferation and viability. Briefly, 10,000 cells were seeded per well in a 96-well plate and allowed to develop for 12–24 h. ROR1 inhibitors dissolved in DMSO (final concentration <0.5%) were serially diluted into the cells in each column, except for the last, which was saved for the DMSO vehicle control group used in all analyses. After 72 h, the treatment media was replaced with fresh untreated media. The MTT dye was applied and incubated at 37°C for 2 h. Formazan crystals were dissolved in DMSO. Finally, the absorbance was measured at 540 nm. Cell viability was evaluated as a percentage of untreated control cells. GraphPad Prism software (version 8.0) was used to calculate IC_50_ values.

### Apoptosis assay

To assess apoptosis after treatment with ROR1 inhibitors, Caspase 3/7 staining was performed using the Muse Caspase 3/7 assay kit (Luminex MCH100108). The cells were seeded at a density of 250,000 cells/well in a 6-well plate. The cells were treated with either ROR1 inhibitors at the IC_50_ or DMSO vehicle control for 72 h. Next, the cells were trypsinized, resuspended in an assay binding buffer, and stained with Caspase 3/7 to be analyzed for apoptosis using a Muse Cell Analyzer.

### Immunoblotting

After ROR1 inhibitor treatment (10 µM) and vehicle (DMSO) control treatment, the cells were collected by scraping. The proteins were lysed using RIPA buffer (150 mM NaCl, 1.0% Triton-X-100, 0.5% sodium deoxycholate, 0.1% SDS, 50 mM Tris, pH 8.0) with 1 mM PMSF (Protoc, 2017). Bradford Assay measured cell lysates’ protein concentration using Pierce 660 nm reagent (Thermofisher, #22660). The Protein lysates were processed by mixing with laemmli buffer at a 1:1 ratio, followed by boiling. Equal amounts of proteins were loaded into the wells of an SDS-PAGE gel, followed by electrophoresis. The proteins were transferred to a nitrocellulose membrane, then blocked for 30 min in 5% bovine serum albumin. The membrane was incubated overnight at 4°C in anti-ROR1 antibody (Cell signaling, #D6T8C), then washed with TBST (20 mM Tris, 150 mM NaCl, 0.1% Tween 20), then incubated in horseradish-peroxidase-linked anti-Rabbit IgG (Invitrogen, #65-6120) for 1 h at room temperature. Protein bands were imaged by chemiluminescence. GAPDH (Cell signaling, #D16H11) or β-actin (Santa Cruz Biotechnology, #sc47778) were loading controls.

### Statistical analysis

Unless otherwise specified, all data are presented as the mean ± standard error. Statistical significance between groups was determined using a two-tailed Student’s *t*-test or nonlinear regression, with *p*-values less than 0.05 considered significant. All statistical analyses were performed using GraphPad Prism 7.0. (GraphPad Software Inc., San Diego, CA, United States).

## Results

Two sections of PDB, 6TU9, were missing at the beginning and end of the structure. The other two were missing amino acids from 569 to 586 and 648–651. All the sections were homology modelled with the MODELLER plugin in the Chimera visualization tool. A library of 70 thousand compounds was prepared for docking with the ROR1 kinase domain (PDB ID: 6TU9). After docking and consensus voting, 10,000 compounds remained. These compounds were filtered using Python scripts to eliminate structural alerts and PAINS. After this process, 250 compounds were clustered, and the top 10 compounds with the lowest LogP were chosen (Table 1).

**TABLE 1:** After clustering, representative compounds are shown with their docking energies and logP values. The table is arranged in an increasing Glide docking score. After manual visualization, compounds with serial numbers 2 and 4 were selected for bioassay studies.

S. No.	PubChem CID	Glide XP score	AutoDock VINA (1) (kcal/mol)	AutoDock VINA (2) (kcal/mol)	LogP
1	24970243	−9.208	−10.5	−11.0	4.07
2	135399549	−8.551	−10.5	−10.6	2.19
3	2108613	−7.589	−10.9	−10.8	2.57
4	1261330	−6.635	−10.8	−10.8	4.15
5	2952584	−6.498	−10.4	−10.5	3.43
6	2582454	−6.41	−10.4	−10.6	3.37
7	3242931	−6.06	−10.8	−10.8	4.33
8	141381336	−5.482	−10.4	−10.5	3.08
9	655402	−2.732	−10.9	−11.1	2.61
10	648475	−2.404	−10.6	−10.8	1.95

### Structure preparation, docking, consensus voting, and clustering to find the top hits

**FIGURE 1:**
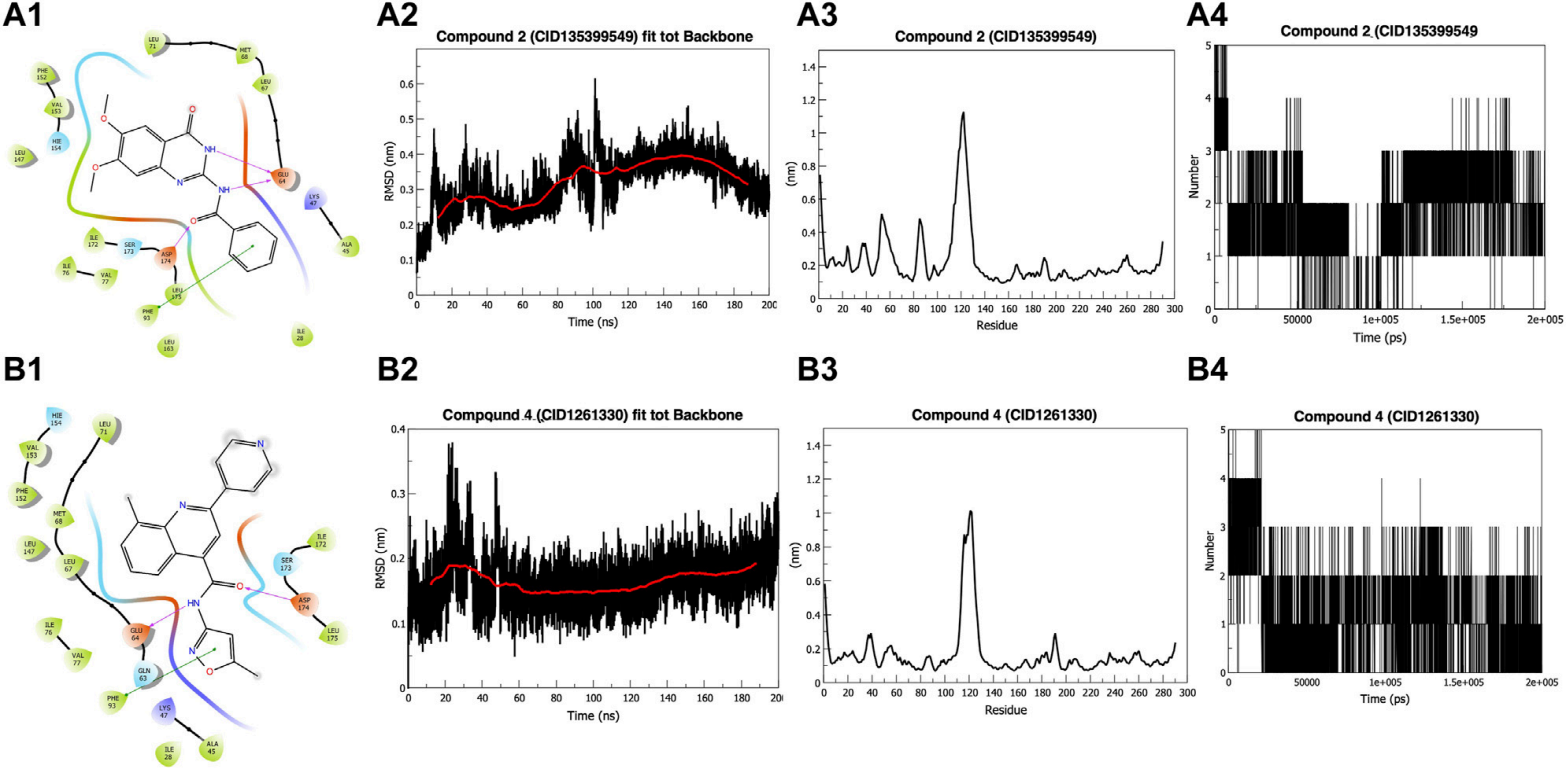
Docking and Simulation. **(A1)** Compound 2 (CID135399549) **(B1)** Compound 4 (CID1261330). Both compounds fit into the pocket and show H-bonding with GLU64 and ASP174 and pi-pi interaction with PHE93 **(A2)** Root Mean Square Deviation (RMSD) of Compound 2 (CID135399549). The compound shows stability around 0.30 nm in the last 100 ns **(B2)** RMSD of Compound 4 (CID1261330). The compound showed stability below 0.20 nm in the last 150 ns **(A3)** Root Mean Square Fluctuations (RMSF) graph of Compound 2 (CID135399549). Do not affect the amino acids 42–65 and 80–85, which are part of the binding pocket **(B3)** RMSF graph of compound 4 (CID1261330). It showed stability with binding pocket residues **(A4)** Hydrogen-bond graph of compound 2 (CID135399549). Non-consistent H-binding **(B4)** Hydrogen bond graph of compound 4 (CID1261330). Two stable bonds were formed during the simulation.

On manual inspection, we discovered that compound 9 (CID655402) interacted with LYS47, but compound 10 (CID648475) did not (Supplementary Figures S7, S8). Both compounds did not fit into the kinase pocket and were exposed to the outer polar environment. Furthermore, compound 8 (CID141381336) (Supplementary Figure S6) partly fits into the pocket, establishing a pi-pi stacking interaction with residue HIE154 but no hydrogen bond. Compounds 6 (CID2582454) and 7 (CID3242931) (Supplementary Figures S4, S5) establish hydrogen bonds with GLU64 and ASP174, as well as a pi-pi interaction with PHE93, whereas compound 5 (CID2952584) (Supplementary Figure S3) creates two pi-pi stacking with PHE93. Three compounds (6, 5, and 7) were more stable than previously outlined. Compounds 2 (135399549) and 4 (1261330) (Figures 1A1, B1), 3 (CID2108613), and 1 (CID24970243) (Supplementary Figures S1, S2) fit into the kinase pocket, show H-bonding with GLU64 and ASP174, and pi-pi stacking with PHE93, demonstrating type 2 inhibitor characteristics (Roskoski, 2016; Martinez et al., 2020; Zhao and Bourne, 2020). Compound 1 (CID24970243) formed an extra hydrogen bond with GLU60, providing additional stability to the ligand. PHE93, ASP174, and GLU64 are essential at the binding site, as PHE93, part of the hinge region, forms a pi-pi bond with seven compounds. Six compounds make hydrogen bonds with ASP174, a component of the DLG region, and GLU64, a component of the C helix (Sheetz et al., 2020).

### Molecular dynamic simulations for the stability of compounds in the kinase pocket

The Root Mean Square Deviation (RMSD) value of compound 2 (CID135399549) fluctuates for the initial 15 ns to reach 0.35 nm and then gets a stable trajectory for 85 ns at roughly 0.25 nm. Further, the structure stabilizes for the remaining 100 ns around 0.30 nm (Figure 1A2). In contrast, compound 4 (CID1261330) exhibited stability after 50 ns, below 2 nm (Figure 1B2). The starting, ending, and loop regions of the root mean square fluctuation (RMSF) graph exhibit high fluctuations, which is expected due to less rigidity. Comparing the two compounds, we found that compound 2 (CID135399549) did not affect the amino acids of the protein from 42 to 65 and 80 to 85, which are parts of the binding pocket, whereas compound 4 (CID1261330) showed stability, as the fluctuations in the binding pocket residues were comparatively less. (Figures 1A3, B3). For compound 4 (CID1261330), we observed a maximum of five hydrogen bonds at the beginning of the simulation, and the two bonds remained constant. (Figures 1A4, B4). Compared to compound 2 (CID135399549), compound 4 (CID1261330) had stable trajectories and more interactions with the binding pocket. Our *in silico* results indicated that compound 4 (CID1261330) is expected to be potent against malignancies expressing high levels of ROR1.

### Cell viability and apoptosis assay to test the inhibition activity of compound 4

**FIGURE 2:**
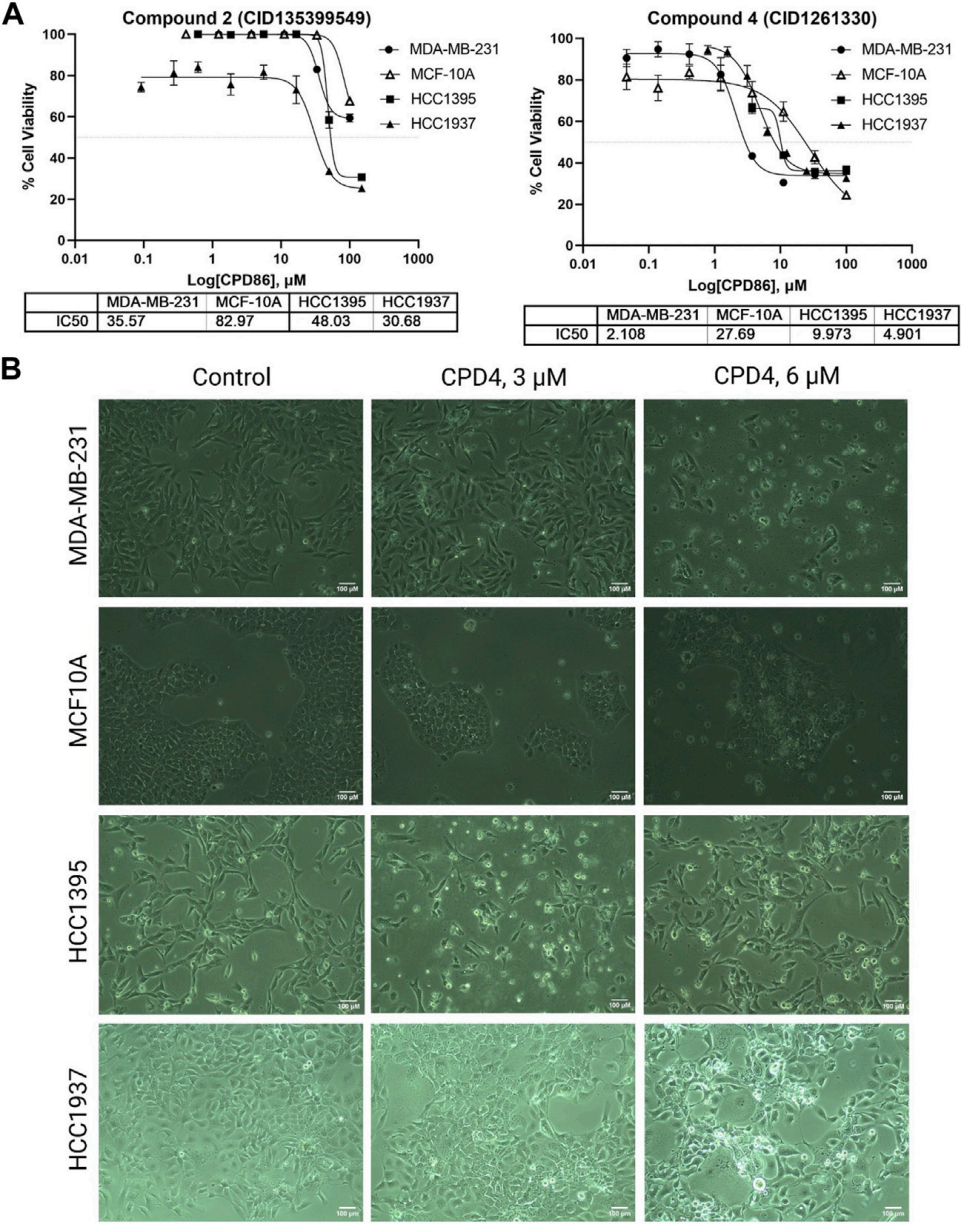
Compound 4 (CID1261330) suppressed TNBC cell proliferation. **(A)** Cell viability assays to assess the IC50 values of Compound 2 (CID135399549) (left) and Compound 4 (CID1261330) (right) of three TNBC cell lines (MDA-MB-23, HCC1395 and HCC 1937) and a non-malignant MCF-10A line (*n* = 3 biological replicates). **(B)** Morphology of the three cell lines after treatment with compound 4 (CID1261330) or vehicle control (0.3% DMSO) at ×10 magnification post 72 h treatment.

Compound 4 (CID1261330) inhibited proliferation and induced apoptosis of TNBC cells. The potency of ROR1 inhibitors compounds 4 (CID 1261330) and compound 2 (CID135399549) toward TNBC cells (MDA-MB-231, HCC1395, HCC 1937) was assessed using the MTT cell viability assay. Compound 4 (CID1261330) significantly suppressed cell proliferation in the TNBC cell lines, MDA-MB-231 (with high ROR1 expression), HCC1937 and HCC1395, at a low half-maximal inhibitory concentration (IC_50_) of approximately between 2 μM and 10 μM, respectively. Non-malignant epithelial breast cells MCF-10A were markedly unsusceptible to compound 4 (IC_50_ > 27 μM) (Figure 2A, right). The cell viability results were consistent with the morphology of the cell post 72 h treatment with the compound. The TNBC cells (MDA-MB-231, HCC 1937; HCC 1395) showed apoptotic vesicles and dead cells post 72 h treatment, while MCF10A did not show any cell death (Figure 2B). This result suggests that compound 4 (CID1261330) is potent and selective in impairing the growth of TNBC cells with minimal damage to normal cells. In contrast, compound 2 (CID135399549) showed no selective inhibitory effect on TNBC cell proliferation (Figure 2A, left). Therefore, Compound 4 (CID1261330) was selected for apoptosis screening.

**FIGURE 3:**
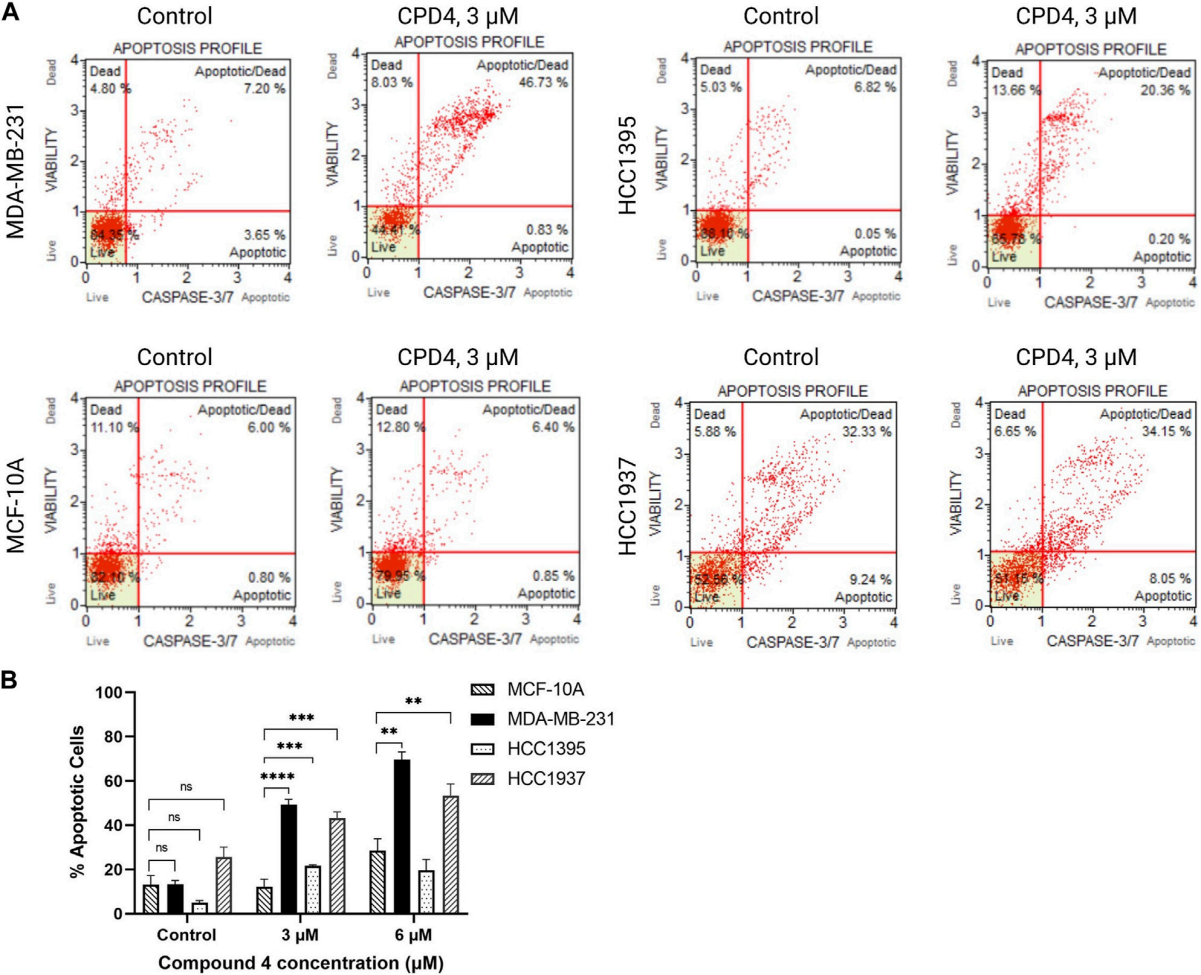
.Compound 4 (CID1261330) at a concentration of 3 μM induced apoptosis in TNBC cells post 72 h treatment. **(A)** Caspase 3/7 staining and 7-AAD assay to assess apoptosis in the four cell lines after treatment with compound 4 (CID1261330). **(B)** Quantification of apoptosis induced by compound 4 (CID1261330) (total of top and bottom right quadrants) in three TNBC cell lines and normal MCF-10A cells (**p < 0.01, ***p < 0.001, ****p < 0.0001, *n* = 1–3 biological replicates).

ROR1 activity induces cancer cell survival and inhibits apoptosis (Zhang et al., 2012; Zheng et al., 2016). Therefore, flow cytometry experiments were conducted to assess apoptosis induction after 72-h treatment with compound 4 (CID1261330). Compound 4 (CID1261330) treatment induced a markedly high proportion of apoptotic MDA-MB-231 cells (47.6%), whereas minimal apoptosis to normal breast cells MCF-10A (7.3%) (Figure 3). The apoptosis results revealed compound 4 (CID1261330) induced dose-dependent apoptosis in MDA-MB-231 cells but not non-malignant MCF-10A cells.

### Compound 4 inhibits the expression of ROR1 in TNBC cells

**FIGURE 4:**
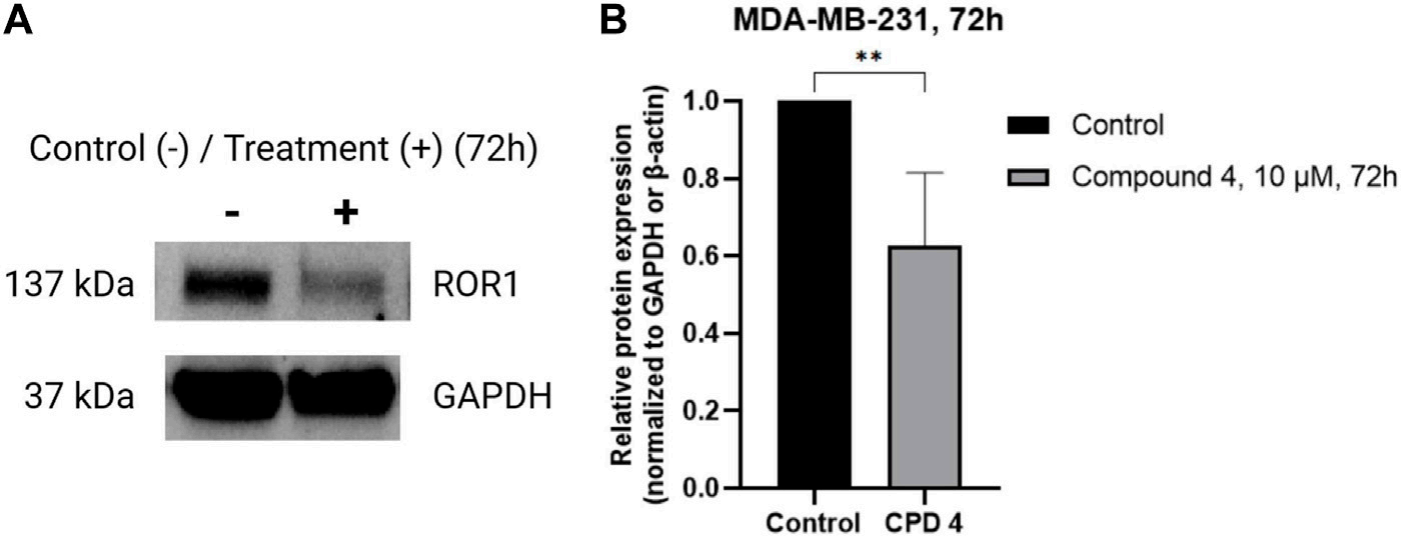
Compound 4 inhibits ROR1 expression levels of TNBC cells. **(A)** Representative western blots of ROR1 on MDA-MB-231 cells treated with vehicle and 10 μM of Compound 4 for 72 h. **(B)** Quantifying panel A immunoblot bands of ROR1 normalized to GAPDH or β-actin. (**p < 0.01, Similar trends were observed in *n* = 3 biological replicates, Supplementary Figure S1).

To determine if compound 4 targets ROR1, MDA-MB-231 cells were treated with 10 μM of Compound 4 for 72 h, and immunoblots were performed in whole cell lysates. Figure 4 shows that Compound 4 reduced the expression levels of ROR1 in TNBC cells by at least 4-fold.

## Discussion

Current therapeutic agents for TNBC, such as anthracyclines, taxanes, or capecitabine, are cardio-, neuro-, and hepato-toxic to patients, causing a host of disastrous side effects and poor prognosis (Cella et al., 2003; Yoldas et al., 2019; Fortes et al., 2022). Therefore, developing a novel targeted anti-TNBC therapeutic candidate with negligible effects on normal tissues is crucial. Many cancers, including breast cancer, have an overexpression of the ROR1. Epithelial-mesenchymal transition, which explains cancer invasion, metastasis, and progression, was found to be mediated by mediators such as ROR1 (Abdelbary et al., 2022). ROR1’s extracellular domain binds to Wnt5a (Fultang et al., 2019; Kipps, 2022). The interaction between ROR1 and various proteins leads to the formation of a complex, resulting in the activation of these proteins. This activation enhances the migration of chronic lymphocytic leukemia cells, which is strongly associated with poor overall cancer survival (Peng et al., 2022). In addition, research has demonstrated that ROR1 plays a crucial role in the glucocorticoid receptor-induced lung metastatic colonization process. Silencing ROR1 has been shown to reduce the spread of metastasis and improve survival rates in breast cancer patients (Menck et al., 2021). Moreover, another study has revealed the existence of a signaling axis involving ROR1, HER3 (ERBB3), and a long noncoding RNA (lncRNA) (Li et al., 2017). This axis has been found to enhance bone metastasis in breast cancer by modulating the Hippo-YAP pathway (Meng et al., 2021).

Several studies have consistently demonstrated that ROR1 exhibits limited kinase activity, primarily due to specific changes in highly conserved amino acids within its kinase domain’s GXGXXG motif. These alterations result in an inaccessible ATP-binding domain and the inactivation of the activation loop. Nevertheless, through the use of a kinase-dead mutation (K506A) and an autophosphorylation site mutation (YXXXYY to FXXXFF) in ROR1, researchers have discovered that the kinase domain of ROR1 still possesses crucial signaling capabilities, even in the absence of ATP binding. Interestingly, this modified ROR1 lost its ability to drive the growth of BaF3 cells. Moreover, constitutive phosphorylation of ROR1 has been observed in chronic lymphocytic leukemia (CLL) cells, indicating that ROR1 possesses inherent kinase activity (Hojjat-Farsangi et al., 2013; Zhao et al., 2021). Based on these findings, we selected the intracellular kinase domain of ROR1 for our study.

The current study aimed to identify a potential compound using an *in silico* approach and validate the compounds using *in vitro* experiments. The validation shows the utility of the *in silico* pipelines. There are examples like; Gefitinib for non-small-cell lung cancer (NSCLC) (Muhsin et al., 2003); Erlotinib for NSCLC and pancreatic cancer (Grünwald et al., 2003); Sorafenib for renal, liver, and thyroid cancer (Wilhelm et al., 2006); and Alpelisib and Lapatinib for breast cancer (Wood et al., 2004; Markham, 2019) which were developed using the *in silico* approaches (Salman et al., 2021). Researchers should analyze interactions in terms of structural and energetic parameters to find the best compounds (Bissantz et al., 2010).

We focused more on the protein-ligand interaction than the docking energy. Docking energy needs to be used as the filtration criteria, not the selection criteria (Ramirez and Caballero, 2016). Hydrogen bonds, aromatic hydrogen bonds, pi-pi bonds, pi-cation bonds, ionic bonds, water bridges, clashes, and the binding orientation of ligands with protein are more critical (Ban et al., 2017; Bharatam, 2021). Simply docking will not provide information regarding the best-generated pose’s stability; it requires molecular dynamic simulation’s help for further confirmation (Cui et al., 2020). The average Root Mean Square Deviation (RMSD) for a stable ligand must be between 1.5 and 3A. The lower the value, the more stable the docked pose. Good pocket fitting, negative binding energies, stable interactions, and stable MD trajectories increases the prediction quality (Fischer et al., 2021).

The *in silico* approach helped us filter down the 70000 compound datasets to a few hits and further narrowed it down to Compound 4 (CID1261330) following MD simulations. In contrast, compound 2 (CID135399549) showed a better docking score but poor stability in the binding pocket during MD simulations. The apoptosis test results supported this prediction. The primary reason for this could be the weak interaction of compound 2 with the ROR1 intracellular kinase domain and the presence of off-targets. Upon searching PubChem, BioAssay AID1768 showed that compound 2 targets Multiple Endocrine Neoplasia 1 (MEN1) and Lysine Methyltransferase 2A (KMT2A). Off-target compounds may cause cell death in all cell lines, impair average cell growth, and have low potency, making the compound non-selective for ROR1.

## Conclusion

This study demonstrated that compound 4 (CID1261330) appears effective in limiting TNBC cells, inhibiting cell viability, and inducing apoptosis in TNBC cells (MDA-MB-231 and HCC1395 cells) at a half-maximal inhibitory dose of approximately 2–10 µM. We performed the same experiments in the non-tumorigenic cell line MCF10A (normal breast epithelial cells with minimal ROR1 expression) and observed no significant cytotoxic effects. Further corroborating these data are immunoblots showing that ROR1 in TNBC cells is successfully knocked down by Compound 4 (CID1261330). This suggests that the inhibitory effect of Compound 4 is specific to TNBC cells because of the presence of ROR1 receptors. Hence, we propose that compound 4 (CID1261330) specifically targets TNBC cells with the least harm to normal breast cells. We believe compound 4 (CID1261330) has therapeutic potential for treating TNBC. Our data sets the stage to further validate compound 4 as a ROR1-inhibitor and an anti-tumor agent.

## Data Availability

The original contributions presented in the study are included in the article/Supplementary Material, further inquiries can be directed to the corresponding authors.

## Footnotes

1 Key Statistics for Breast Cancer 2023, American Cancer Society—https://www.cancer.org/cancer/types/breast-cancer/about/how-common-is-breast-cancer.html#:∼:text=It%20is%20about%2030%25%20(or,(DCIS)%20will%20be%20diagnosed
