# Supplementary figures and images for: Identification and validation of a small molecule targeting ROR1 for the treatment of triple negative breast cancer
Source: Front Cell Dev Biol. 2023 Sep 13;11:1243763. doi: 10.3389/fcell.2023.1243763 (PMC10534069; doi:10.3389/fcell.2023.1243763)

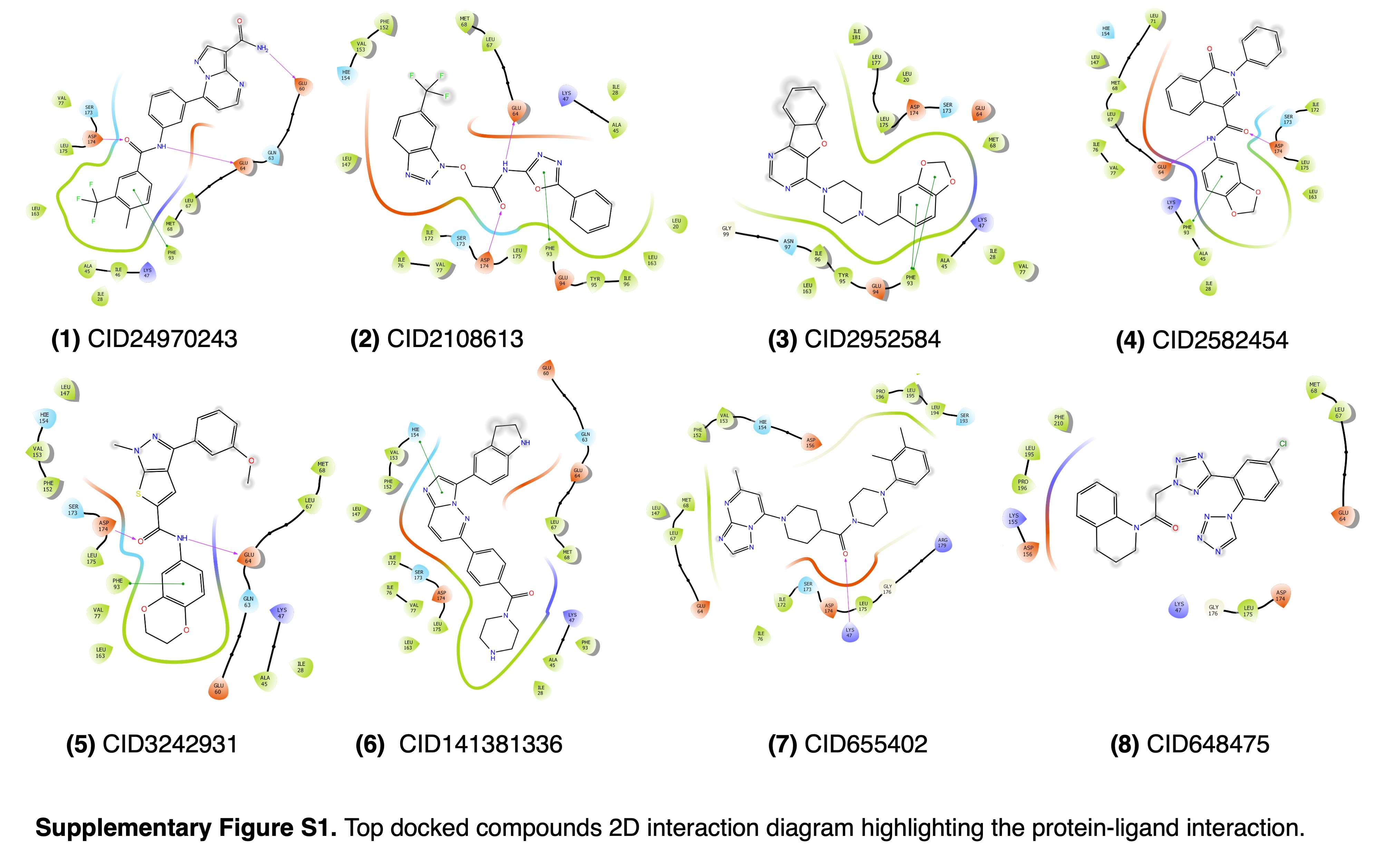

Supplement: Supplementary file 1 [file Image1.JPEG]
