# Supplementary material for: Identification and validation of a small molecule targeting ROR1 for the treatment of triple negative breast cancer
Source: Front Cell Dev Biol. 2023 Sep 13;11:1243763. doi: 10.3389/fcell.2023.1243763 (PMC10534069; doi:10.3389/fcell.2023.1243763)
Supplement: Supplementary file 2 [file DataSheet1.PDF]

Supplementary Data 1:

1. Lanes visualized in the manuscript are labeled. Unlabeled lanes were not used.

2. All proteins used in the manuscript and used for analysis are appropriately labeled. Unlabeled proteins were either not used for analysis or were labeled in subsequent slides.

3. Membranes that were incubated with HRP conjugated secondary antibodies were visualized using a Biorad chemiluminescence image.

4. “MDA-MB-231 control (-)” and “MDA-MB-231 treatment (+) ” labeled lanes are proteins from cells treated with vehicle or 10 µM of Compound 4 for 72 hours, respectively.

5. All densitometry analysis was normalized to housekeeping proteins (GAPDH or b-actin).

11/09/2022

MDA-MB-231 cell line

Control (-) / Treatment (72h)

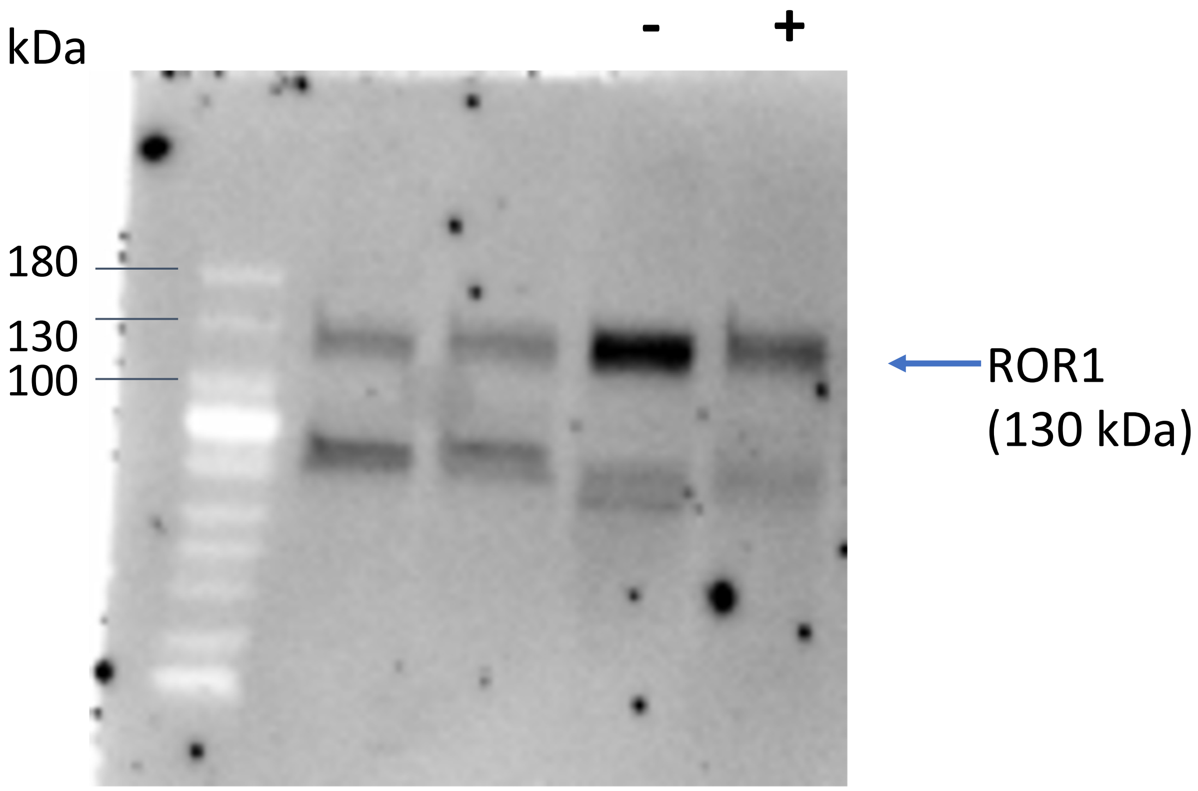

MDA-MB-231 cell line

Control (-) / Treatment (72h)

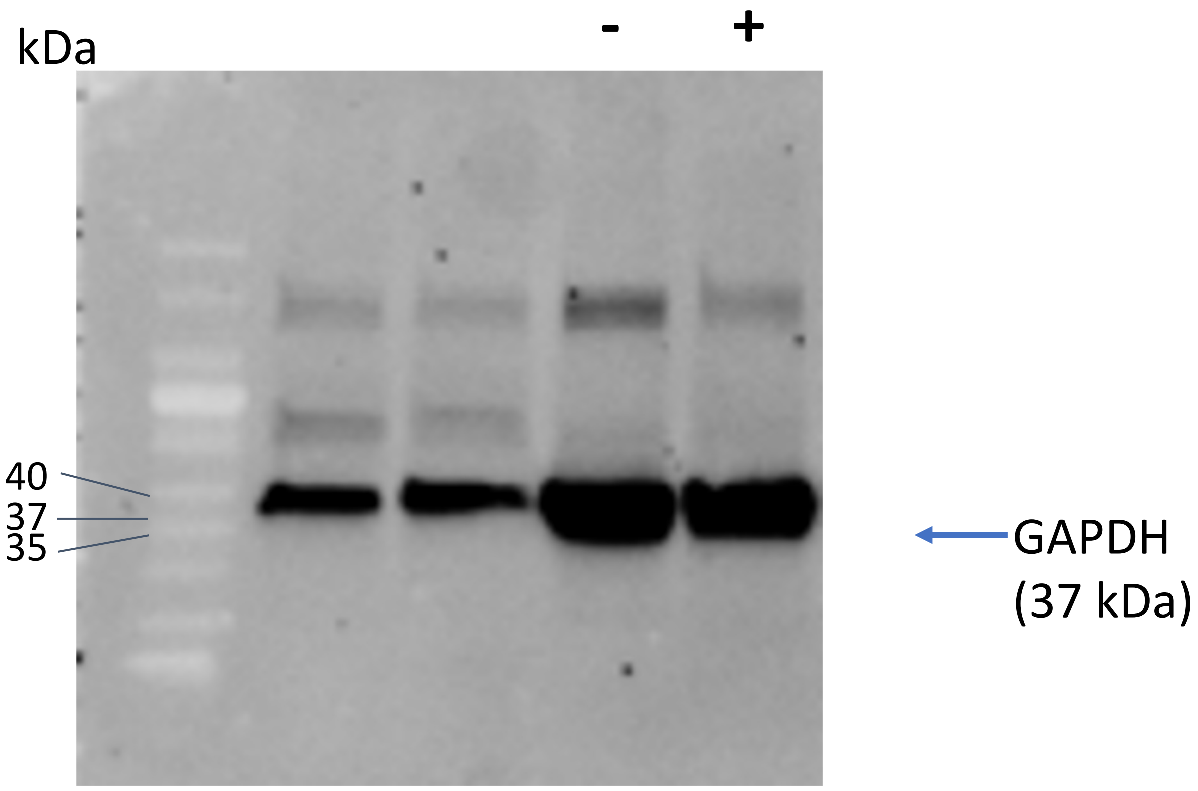

Western blot images from this replicate were used in Figure 4.

05/30/2023

MDA-MB-231 cell line

Control (-) / Treatment (72h)

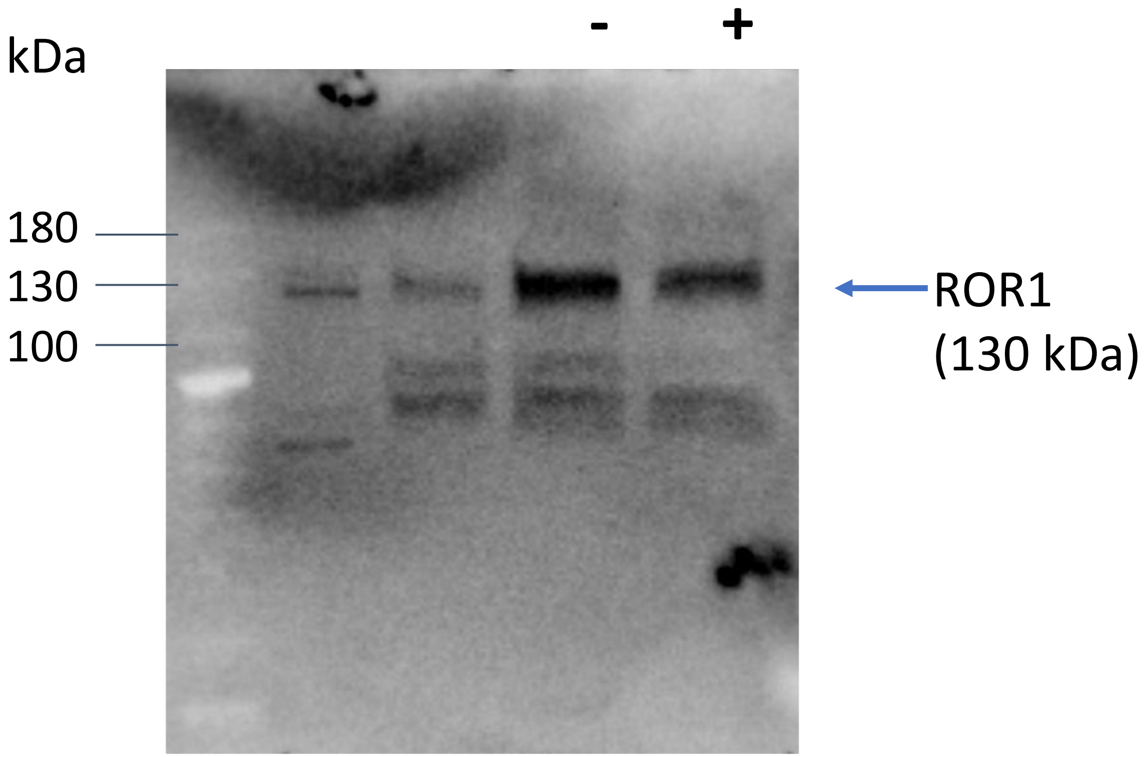

MDA-MB-231 cell line

Control (-) / Treatment (72h)

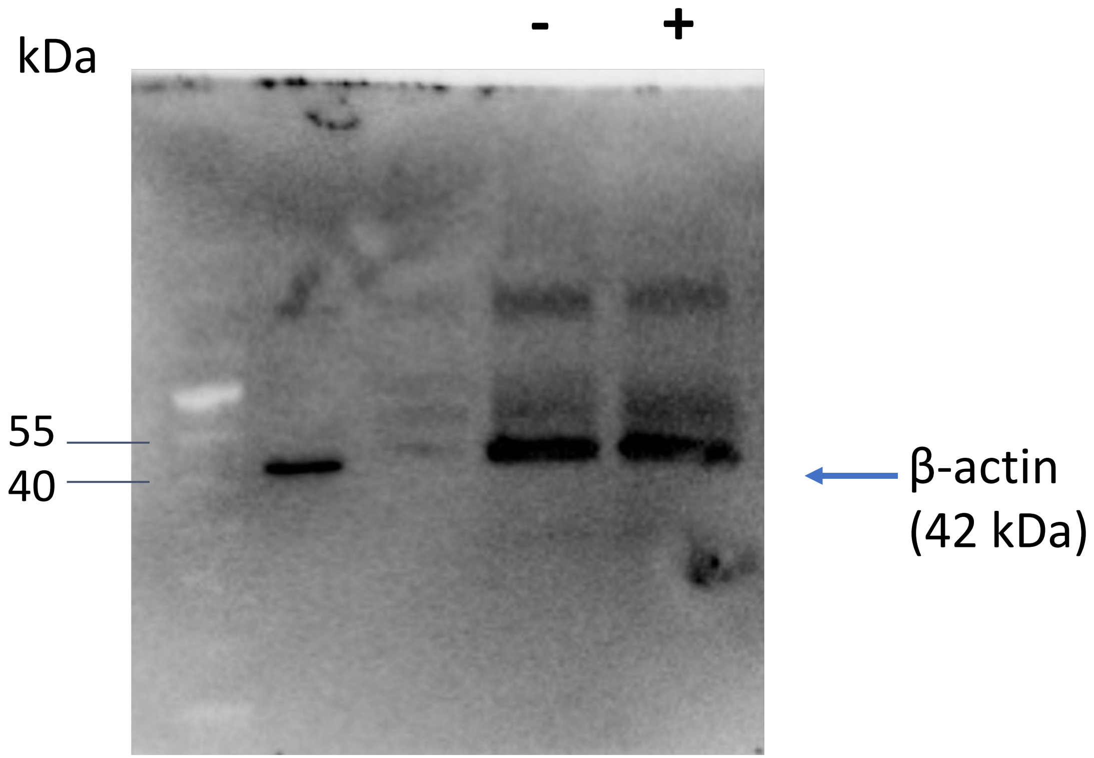

06/01/2023

MDA-MB-231 cell line

Control (-) / Treatment (72h)

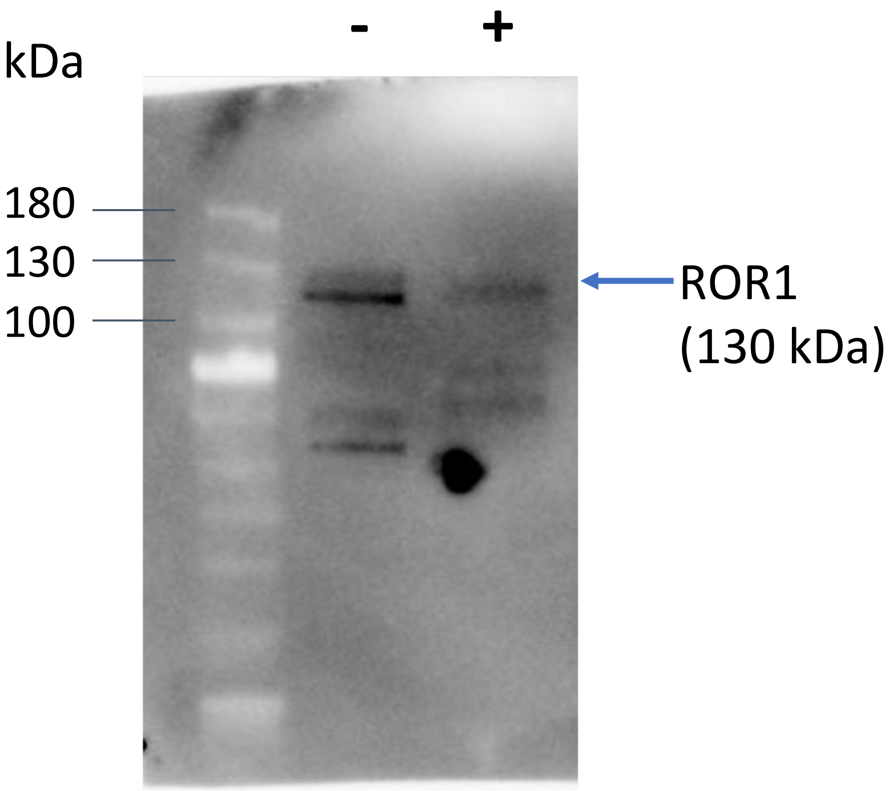

MDA-MB-231 cell line

Control (-) / Treatment (72h)

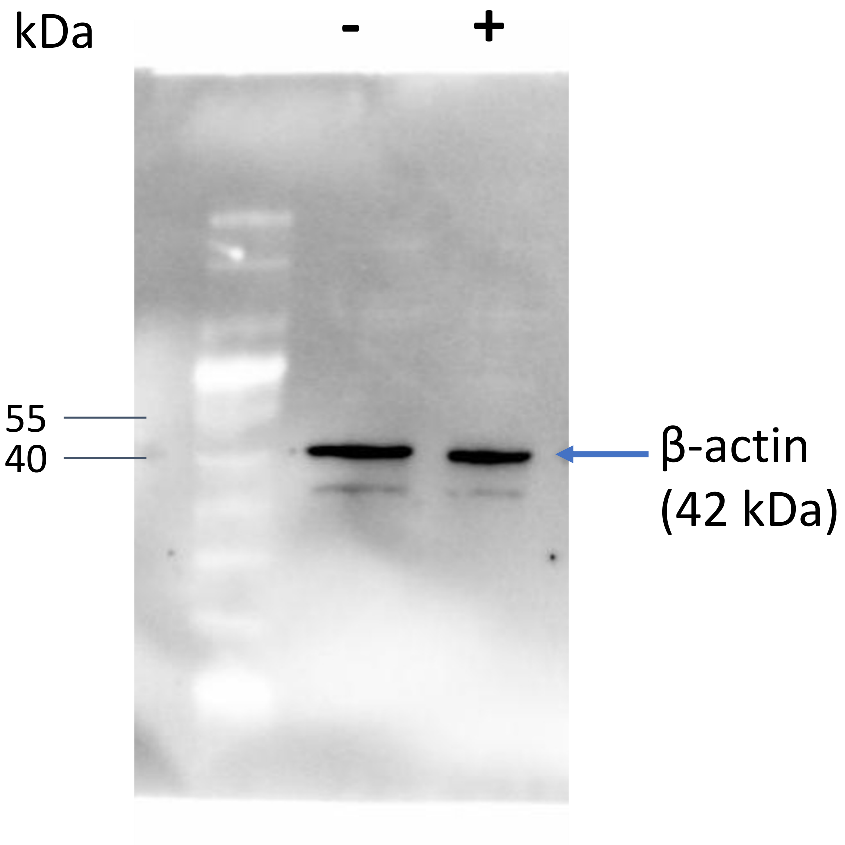

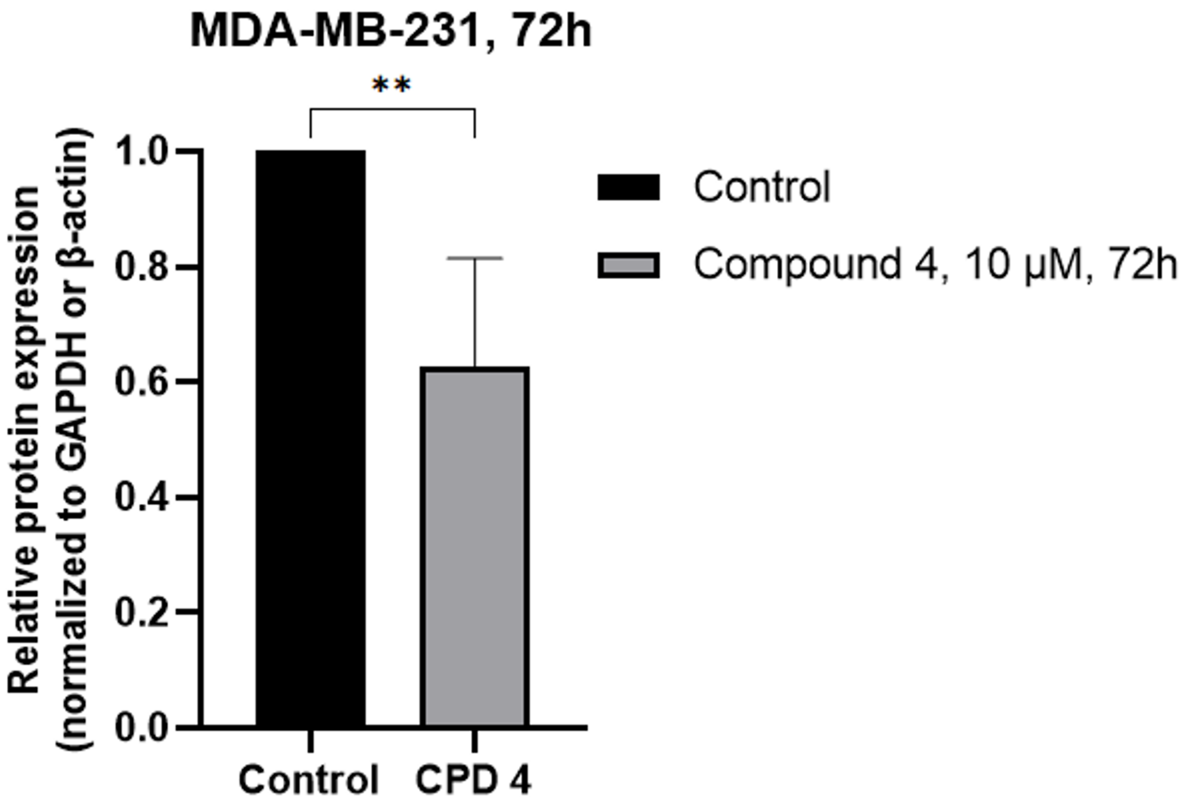

Quantification of immunoblot bands of ROR1, normalized to GAPDH or β-actin. (**$p < 0.01$, n=3 biological replicates).
